# Workshop Report: Governance of Emerging Nanotechnology Risks in the Semiconductor Industry

**DOI:** 10.3389/fpubh.2020.00275

**Published:** 2020-07-07

**Authors:** Ponnapat Watjanatepin, Valentina Castagnola, Yüksel Cetin, Igor Linkov, Claire Skentelbery, Dimiter Prodanov

**Affiliations:** ^1^Environment, Health, and Safety, Imec, Leuven, Belgium; ^2^Centre for BioNano Interactions (CBNI), UCD, Dublin, Ireland; ^3^Genetic Engineering and Biotechnology Institute, TUBITAK, Kocaeli, Turkey; ^4^US Army Engineer Research and Development Center, Concord, CA, United States; ^5^Engineering and Public Policy, Carnegie Mellon University, Pittsburgh, PA, United States; ^6^Nanotechnology Industry Association, Brussels, Belgium

**Keywords:** nanotecehnology, risk assessment, risk communication, semiconducors, policy recommendation

## Abstract

Assessment of risk in the field of nanotechnology requires an integrated multidisciplinary approach due to the complex and cross-disciplinary framework for materials and activities at the nanoscale. The present paper summarizes the workshop “Governance of emerging nano-risk in the semiconductor industry” held on April 26, 2018 in Brussels, Belgium. The event targeted representatives of stakeholder communities involved in the risk assessment and governance of the engineered nanomaterials. Nanoelectronics was selected as an impactful use case for risk assessment approaches and comparison to bottom-up nanofabrication. The workshop outlined key data gaps impeding successful assessment of risks associated with nanoparticle use in the industry, using the semiconductor industry as an example. The workshop outlined mitigation strategies informing future regulatory decisions and identified some directions for future efforts.

## Introduction

Nanotechnology involves a growing number of industrial applications with a large actual economic impact. An increasing number of engineered nanomaterials (ENMs) are entering the global market via consumer products—from healthcare and leisure to electronics, cosmetics, energy, agriculture, food, and transport (see http://nanodb.dk/en/analysis/consumer-products/#chartHashsection). While for bulk substances there are established regulatory frameworks dealing with the risk for the consumers, workers, and the environment (1), this is not the case for nanomaterials. This situation has created a vibrant nanosafety research community, with the primary objective of ensuring that society is able to use nanomaterials safely and with confidence. It is generally accepted that the recently developed and upcoming large variety of nanomaterials presents a challenge for assessing associated risks using the traditional chemical risk assessment methods. Therefore, a multitude of new risk assessment tools has been developed by researchers (2–6).

Acceptance and subsequent wider use of one or another risk assessment framework will inevitably have a wide range of economic and safety repercussions and, therefore, is of considerable worldwide science policy interest. For example, in the European Union, nanosafety research has been increasingly supported throughout the Framework Programmes (FP) 6 and 7 and Horizon 2020. Whereas, only five nanosafety projects were funded in the 6th Framework Programme (2002–2006), in the 7th EU Framework Programme around 50 projects and initiatives on nanosafety were funded between 2007 and 2013. By 2018, 19 projects were funded under H2020 (7). Furthermore, in order to increase the sharing of information and avoid duplication of efforts, nanosafety-related projects cooperate within the framework of the EU NanoSafety Cluster, an informal coordination program supported by the European Commission Directorate-General for Research and Innovation (DG RTD). In the USA, the National Nanotechnology Initiative (NNI, www.nano.gov) coordinates 20 departments and independent agencies working together toward creating a framework for shared goals, priorities, and strategies that helps each participating Federal agency leverage the resources of all participating agencies toward nanotechnology innovation. Environmental Health and Safety is governed by the Nanotechnology Environmental and Health Implications (NEHI) Working Group, which develops Environment Health and Safety Research Strategy (https://www.nano.gov/node/681). Specifically, for occupational health and safety relevant to the semiconductor industry, the National Institute of Occupational Safety and Health (NIOSH) provides guidance on the occupational safety and health implications and applications of nanotechnology (8). NIOSH has identified 10 key research directions informing policymaking choices. One of these priority areas is risk assessment focusing on two research directions: utility of current exposure-response data for identifying potential occupational hazards and development of predictive frameworks for risk and evaluation of hazards. NNI and EU Nanosafety Cluster coordinate activities through the US-EU communities of research (https://us-eu.org/communities-of-research/). Commonly organized activities include thematic workshops and joint publications.

The international regulatory science efforts are coordinated by the Organization for Economic Co-operation and Development (OECD) at the level of the Working Party on Manufactured Nanomaterials (WPMN).

Assessment of risk related to nanotechnology innovation requires an integrated multidisciplinary approach due to the complex and cross-disciplinary framework for materials and activities at the nanoscale. Notably, as a minimum, such disciplines could be material science, aerosol physics, occupational medicine, toxicology, chemical technology and process safety. It should also be noted that partially because of the vigorous science policy interest—the number of publications presenting data on the toxicology of nanomaterials has increased tremendously, although most studies are not directly applicable at the industry level for risk assessment of ENM (9, 10).

ENMs represent bottom-up nanotechnology, in which particles, fibers or nano-surfaces are synthesized from molecules. In contrast, top-down nanotechnology can be exemplified by the semiconductor lithographic processes, where nano-scale features are patterned with exceptional precision on macroscopic objects, such as 300 mm wide silicon wafers. Integration of bottom-up and top-down approaches is a challenge that needs to be overcome (11).

Nanotechnology is expected to become a key pillar for a European economy driven by innovation; therefore, it is very important to foster an open culture of communication of identified hazards and risks to maximize the industry's socio-economic impact. The present paper summarizes the workshop “Governance of emerging nano-risk in the semiconductor industry” held on the April 26, 2018 in Brussels, Belgium. The event targeted representatives of stakeholder communities involved in the risk assessment and governance of ENMs. The workshop was co-organized by the NanoStreeM and caLIBRAte H2020 projects with the support of the Flemish Royal Academy for Arts and Sciences (KVAB).

Conferences and workshops related to nanosafety are organized on a regular basis in Europe. The general co-ordination forum for all nano-safety related project are the EU NanoSafety Cluster events, which are organized twice a year. An important policy forum organized by the European Commission is the EuroNanoForum, held bi-annually. International co-operation is maintained by the EU-Asia Dialogues in nanosafety and the EU-US Communities of Research workshops.

## General Remarks

The program of the workshop was organized into three sessions:

1. The use of engineered nanomaterials in the semiconductor industry.
2. Challenges in the current risk assessment, including industry needs and technological advances in risk management.
3. Experiences in risk management from the producers and users of nanomaterials.

The event gathered more than 40 representatives of various stakeholder communities. The workshop featured 10 invited speakers and 11 panelists from different backgrounds, including academia, research institutes, industry, and the European Commission. The workshop fostered a culture of open and lively information exchange between the academic participants, policymakers, and representatives of the industry. Throughout the day, several principal challenges were identified:

- There appears to be accelerated growth of ENM entering the market worldwide. This presents both an opportunity for rapid economic development and a potential threat of unanticipated hazards.
- It is difficult to predict the hazards and risks related to the use of new nanomaterials. Because of the exponential growth in the numbers of combinations of new materials and nanoforms, the relative lack of resources makes developing regulatory approaches challenging.
- There is a clear safety knowledge gap, notably in the emission and exposure assessments. Furthermore, toxicity data about nanoforms of nanomaterials are also frequently lacking or are insufficient to determine occupational hazards.
- Many nanotoxicity databases developed to date are either not available for public use or the available data cover only a few materials. For example, OECD prioritized only 11 materials.
- Risk analysis is still technically and methodologically limited. Notably, the available models are very generic, developed at population scales and difficult to adapt for life cycle exposure by the concerned industries.

A more detailed list of challenges can be found in Appendix 1. The open discussion on the floor confirmed that conventional chemical risk assessment methodology is not fully applicable for newly developed materials or nanoforms entering the market. It could be concluded that nanomaterial risk assessment requires specialized knowledge on toxicity testing and strategies, in addition to lifecycle exposure and emissions, which is not readily available at present outside fragmented studies in academic communities. The complexity of the subject and the substantial time lags whenever data becomes publicly available pose a definite regulatory challenge. Approaches for categorizing nanomaterials in similarity groups [i.e., such as hazard bands or classes (12–14)] developed by several EU-funded projects may result in solutions that are practically achievable and acceptable to stakeholders. In another example, the US Consumer Product Safety Commission (CPSC) is using risk-based grouping of nanomaterials to prioritise testing and evaluation of nano-enabled product that are likely to pose consumer safety risks (14). Health and safety assessment can be empowered by the available international standards supported by the International Organization for Standardization (ISO), European Committee for Standardization (CEN), and OECD. Nevertheless, regulatory experience with generic models proposed by these bodies to date does not favor a preference toward one universal risk assessment framework vs. multiple industry/application/material specific risk management tools. It is, therefore, important to develop expert competencies allowing for successful governance of risks using multiple tools.

In order to maximize the socio-economic impact of nanotechnology it is also important to identify appropriate sources of information and foster communication channels for all actors along the supply chain. Risk acceptance along the supply chain depends strongly on the understanding of the chemical or ENM hazards. Therefore, appropriate and efficient communication of the hazards between the different actors along the supply chain is paramount for successful management of emerging risks and hazards. The panelists held the opinion that the available regulatory frameworks, such as the REACH Regulation 1907/2006 (2), are sufficient to communicate the hazards of nanomaterials along the supply chain. It could be also concluded that the labeling of raw nanomaterials could be helpful when suitable communication channels are established.

The panel concluded that specific risk governance approaches should:

(a) identify gaps and uncertainties with decision making that is based on traditional risk assessment,
(b) prioritize such gaps based upon available statutory requirements and industry requirements, among others, and
(c) allow for iterative updating and improvements to regulatory practice as new information on nanomaterial risk becomes available.

## Session Reports

The following sections give a more detailed account of the three thematic sessions.

### Session “Nanomaterials in the Semiconductor Industry”

**Figure 1:**
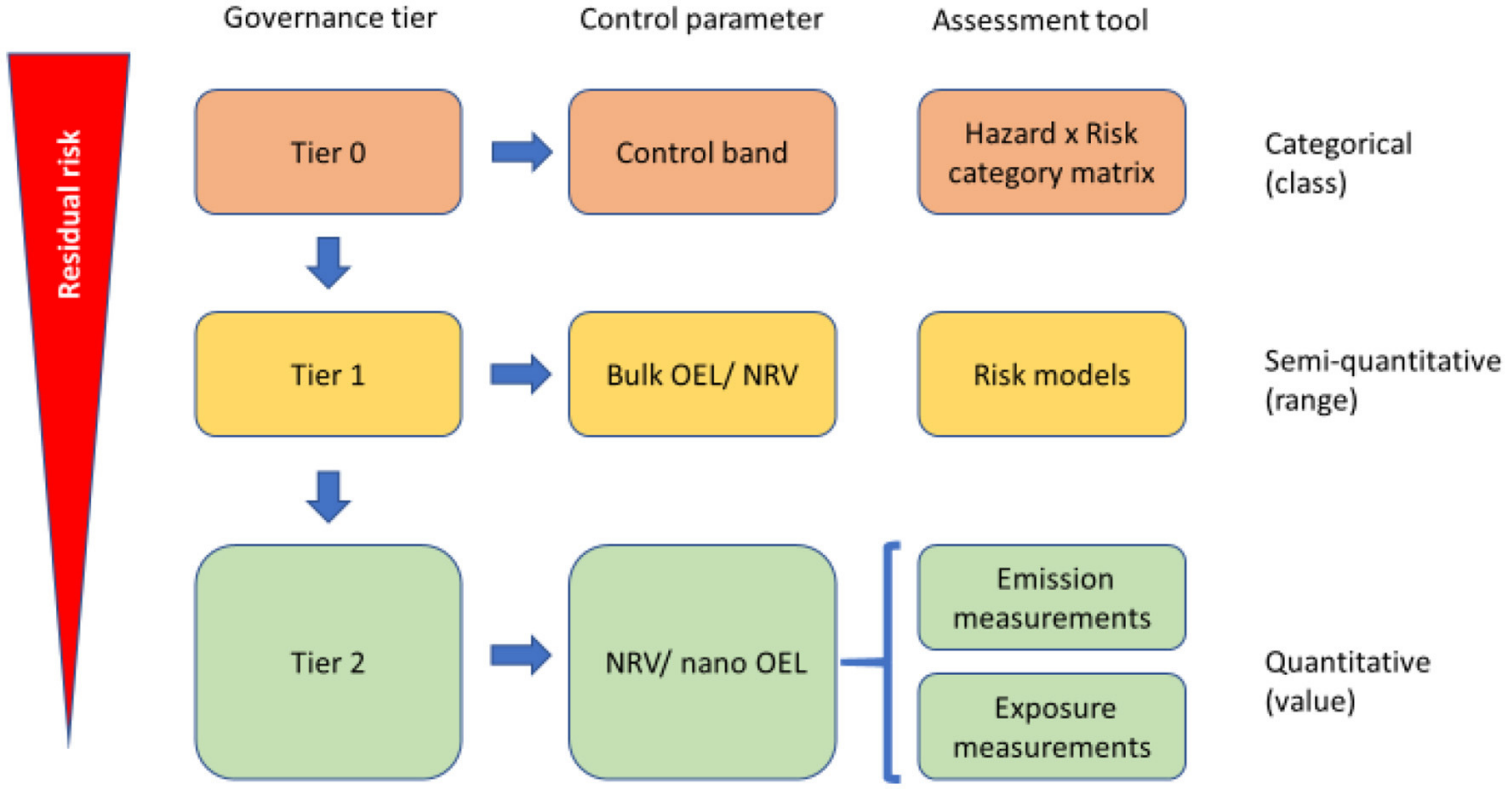
NanoStreeM risk assessment and control methodology (17).

The workshop was opened by the NanoStreeM project consortium with a presentation given by the coordinator, Dr. Dimiter Prodanov (Imec). Dr. Prodanov stated the objectives of the workshop, notably to (i) present how and where nanomaterials are used in the semiconductor industry, (ii) identify appropriate risk control methodologies and their limitations, and (iii) support communication of risk along the supply chain and to society. Dr. Prodanov further introduced the research and development challenges in the semiconductor industry and presented briefly the main findings of NanoStreeM about the use of engineered nanomaterials (ENMs) in the semiconductor industry, given the potential worker exposure (15, 16). In addition, the NanoStreeM tiered risk assessment approach was presented as a starting point for discussion (17) (Figure 1). The presentation showed the main topics of discussion in the workshop, including the main barriers of the current state of risk assessment guidance and tools and whether, based on the current state of knowledge and experience, the effort direction should either be toward a universal risk assessment framework or an industry/application/material specific framework. Subsequently, the key questions which the panels would discuss were also outlined.

The keynote lecture of Prof. Kai Savolainen (FIOH) presented the state-of-the-art in nanosafety and the frameworks which are being adopted to assure the occupational safety of engineered nanomaterials in an industrial setting (18). He started by introducing the relevancy of ENMs in the semiconductor industry, then showed several provisional tools that have been introduced by different agencies, such as the Recommended Exposure Limits (REL) for Nano by NIOSH.

Dr. Fiona Moclair (Intel) subsequently presented the key findings of NanoStreeM about the use of ENM and demonstrated how NanoStreeM has attempted to bridge the current information gaps through its tiered risk assessment approach. Dr. Moclair stressed that nanomaterials used in polishing are essential for chip manufacturing. Her presentation introduced which processes in the semiconductor industry employ nanomaterials and identified how the framework could be used step-by-step. The presentation discussed the nanomaterials risk profile within the semiconductor industry by discussing the framework in view of the use of the ISO control banding tool (19).

Mr. Thies Oosterwijk (TNO) concluded the session with a presentation comparing the main findings of both NanoStreeM and caLIBRAte about risk assessment approaches. For NanoStreeM, a relevant exposure scenario was given along with the tiered risk assessment framework approach (Figure 1) (20). In the case of caLIBRAte, the focus was on the nano-specific tools that match the stakeholder criteria along with the innovation stages. For both projects, the ongoing activities and current gaps were also identified.

### Session “Challenges of Nanosafety: Industry Needs and Technology Advances in Risk Management”

The second session started with a presentation by Mr. Pascal Roquet (STM) giving feedback about the NanoStreeM risk assessment guidance usability. Mr. Roquet started by introducing the semiconductor fabrication process and the necessity of polishing steps, in particular, the Chemical Mechanical Polish (CMP). CMP is a ubiquitous step in all kinds of chip manufacturing technologies (1). During CMP, a wafer is turned face-down on the polishing pad and slurries [e.g., suspensions containing nanoparticles of cerium oxide (CeO_2_), silicon dioxide (SiO_2_), or aluminum oxide (Al_2_O_3_)] are applied during the abrasion process. CMP slurries are a well-established set of products containing nanomaterials that are used within the semiconductor sector. Mr. Roquet discussed the gaps in the current risk assessment approach. Several tools were available but selecting the correct assessment and measurement tools was also a challenge. An example of using the Advanced REACH Tool (21) (ART) and comparing the obtained results with an actual real-time particle emission measurement showed that although ART was not specific to the semiconductor industry, the tool was found suitable for further consideration. He concluded that the assessment and exposure tools were available but not fully calibrated for ENMs specification. Moreover, his presentation pointed out that NanoStreeM has developed a pragmatic list of measurement equipment available on the market, although it was not exhaustive.

The second presentation was given by Prof. Keld Alstrup Jensen (NCWRE, coordinator of caLIBRAte) on global advances in risk assessment and governance. He introduced the current status of the regulatory accepted risk assessment and management models. This presentation identified the main deficiencies of conventional risk assessment approaches and outlined the concept of the Nano Risk Governance Framework developed in the context of the caLIBRAte project and starting from the needs and concerns of the caLIBRAte stakeholders. The presentation concluded by discussing next steps for caLIBRAte and plans for an online Nano Risk Governance Portal, launched in October 2019.

Mr. Joris Quik (RIVM) presented the SimpleBox4nano tool, a multimedia nanomaterial fate model that is adapted for the case of ENMs environmental risk assessment. The model considers several parameters ranging from emission volumes to physicochemical properties to landscape characteristics. A case study of the application of SimpleBox4nano was given with nanoforms of TiO_2_, C60, and Ag. The results indicated that the environmental modeling of nanomaterials was feasible and that the most important inputs were deposition and dissolution rates, attachment efficiency, and particle size.

The final presentation of the session was given by Dr. Gemma Janer (Leitat) on the GuideNano tool, as an example of the industry-focused risk assessment tool. After introducing the conceptual framework, Dr. Janer discussed the GuideNano tool component-by-component. Based on the inputs, the tool would be capable of generating the final exposure estimate, deriving the safety limit value and hazard endpoints, and providing risk mitigation strategy.

### Session “Experiences in Risk Management From the Nanomaterials Producers and Users”

The third session began with a presentation by Mr. Gunther Van Kerckhove (OCSiAl) on the health, safety, and environmental status of TUBALL Nanotubes. Mr. Van Kerckhove started by introducing the unique physicochemical properties of carbon nanotubes (CNTs), which include high conductibility and improved mechanical and dispersion properties. He then discussed safety concerns regarding single-walled carbon nanotubes (SWCNTs), notably their bio-persistency. This can be alarming as bio-persistency could lead to potential carcinogenicity. He demonstrated that, although SWCNTs are registered for wide use in a diverse range of industries, according to REACH and the US Environmental Protection Agency (EPA) there are no sufficient carcinogenicity data at present. Additional studies and tests are planned to produce this missing information.

Subsequently, Dr. Jacques-Aurélien Sergeant (Solvay) presented the experience of Solvay with the hazard banding of ENMs. He addressed three essential questions concerning the use of nanomaterials within Solvay: (1) how to handle the large variety of ENMs within Solvay to ensure safe use, (2) how to handle the large variety of ENMs within Solvay to ensure regulatory compliance, and (3) how to consolidate necessary expertise company-wide to assess the large variety of ENMs. Regarding the first question, control banding approaches have been adopted along with the network of cross-discipline expertise, consisting of an industrial hygienist as the first line of support and a toxicologist as the second. As per the second question, Solvay developed a dedicated tool to centralize and shape data into dedicated templates. Finally, international discussions with main stakeholders as well as involvement in an international research project for further expertise development were employed by Solvay to consolidate the necessary expertise.

The final presentation of the third session was given by Dr. Pasqualantonio Pingue (NEST, Scuola Normale Superiore) on the risk management of graphene and semiconductor nanowires in research and development labs. He presented an integrated control banding as well as exposure measurements to evaluate the risk level according to the ISO control banding matrix (12). A case study involving graphene and nanowires was demonstrated (22).

## Actionable Recommendations

By the end of the workshop, several challenges had been identified. The main challenges could be summarized as follows:

– Identifying and characterizing hazards leads to the difficulty in assessing the risk of associated ENMs due to the lack of standardization and validated protocols.
– From this perspective, a knowledge gap can be identified, especially in the toxicological data and exposure scenario data, which may confound and limit current regulatory approaches. Concerning this gap, Dr. Georgios Katalagarianakis (EC) noted that “it is important to elucidate how much uncertainty is acceptable and how much the industry should pay in the process of eliminating it, because if the requirement is too high, there would be no business and innovation would be suppressed”.
– Although many nanotoxicological databases are compiled, most are not accessible to the public or cover only exemplary materials. Examples are the OECD ENM datasets and the eNanoMapper database (accessible from https://data.enanomapper.net/), which is one of the largest data sources currently available on the toxicological properties and used by the European Union Observatory for Nanomaterials EUON^1^ Moreover, not all of the required physicochemical properties and synthesis methods are given, thus constraining the applicability of such databases.
– The available risk assessments are methodologically and technically limited. The adaptation to daily use in an industrial setting is challenging due to a lack of validation. This comes in contrast to purely academic research into novel tools (18).
– The life cycle of nanomaterials and inactivation treatment of nanoparticle-containing waste should also be investigated, as these are some of the main questions asked by customers of ENMs. This important information, as well as the physicochemical properties of the nanomaterials, can enable ENMs to be manufactured according to the principles of safe-by-design. As emphasized by Dr. Katalagarianakis and Prof. Savolainen, safe-by-design was one of the core principles on which many industries and regulatory authorities focus. Additionally, Dr. Skentelbery emphasized that the framework of safe-by-design should be adopted and promoted to aid innovation.

The interactive discussion between the panel and the audience concerning initial questions can be summarized as follows:

1. **What can be adapted to the regulatory framework approach to take the gaps in the availability of required data, notably toxicological and exposure scenario data, into consideration so that the innovative application of these ENMs is not curtailed?**
  The results of the discussions have confirmed that it is not possible to directly incorporate the traditional chemical risk assessment for ENMs. In fact, risk analysis for ENMs requires specialized expertise on nanotoxicology, exposure, and emissions scenarios, as well as the life cycle of the ENMs. This is why nanosafety decision making requires substantial judgment and training. Consequently, many participants agreed on the need for training nanosafety risk experts. According to the semiconductor processing safety expert Mr. Alain Pardon (Imec), in the long run nanosafety risk experts can be trained so that performing the risk assessment for nanomaterials is multidisciplinary, rather than only done by chemical safety experts. Many participants shared this view. In particular, Dr. Moclair (Intel) indicated that EHS professionals should be trained about the specifics of nanomaterials in order to do a risk assessment for nanomaterials. This is because it is not possible to directly transfer the traditional EHS chemical risk assessment to the level of nanomaterials. Dr. Linkov suggested that emerging tools of Multi-Criteria Decision Analysis could support experts and allow integration of technical data and judgments in a coherent and transparent framework (6).
  The inevitable “pacing” problem is associated with the widening gap between technology innovation, EHS data availability, and ability of the regulatory community to act on the data (23). The participants agreed on the possibility of imminent limits to nanotechnological innovations due to this data gap, which is required to perform risk assessment. The discussion reached a consensus that it was of paramount importance that scientific capacity and competencies were developed so that it would become possible to translate the acquired basic science to the required regulatory science. Dr. Katalagarianakis (EC) added that, as the community improves its experience and knowledge of ENMs, society can improve the safety level and reduce uncertainty, but only through innovation. Currently, science policymakers encourage more data gathering and the development of standardized protocols, which takes time. Still, as the scientific community develops the foundation, science can enable us to advance our predictive capability. This information is also essential in order to enable safety-by-design. Until that has been accomplished, different projects and stakeholder groups have derived several dynamic approaches to group similar nanomaterials into hazard bands or classes. These could be tentatively employed as a practical approach alleviating the current uncertainty. The ISO/TS 12901-2:2014 (12, 19) has also been developed as an international standard that could be of particular interest to environment, health, and safety professionals in conducting assessments.
  Based on the discussions, it becomes apparent that a universal risk assessment framework does not exist for ENMs but rather, a risk assessment methodology that is application-specific or industry-specific is more likely to be used. According to one remark from the audience, the results obtained from different risk assessment tools displayed a rather big discrepancy and thus, model validation for different industries or applications was of paramount importance. However, this was also often not possible as each industry or application is specific to each tool. On a similar note, Mr. Oosterwijk (TNO) agreed that it was more realistic to develop industry-specific tools based on standardized scenarios and limited to a certain list of materials. Pascal Roquet (STM) also agreed that fine-tuning the risk assessment for a specific usage must be done to focus on custom settings which could differ from factory to factory. According to Alain Pardon (Imec), the semiconductor industry is unique in terms of exposure conditions. This is because the semiconductor industry uses closed systems with strong exhausts, namely cleanrooms with forced ventilation, to protect the manufactured products. Hence, it is uncertain whether the industry would ever fit into a generalist framework. Finally, Prof. Alstrup Jensen (NCWRE) also expressed the view that it was more important to first observe the process in question and then to define the tools best suited to handle this process. It is a challenge to find one coherent tool; however, it may be possible to create a framework that indicates or cross-bridges certain tools to appropriate application domains.
2. **What are possible actions that upstream developers, suppliers, and formulators can take to communicate with downstream users regarding risk assessment, guidance, and management?**
  Successful and effective management of risks and hazards requires an adequate exchange of information and communication between different actors along the supply chain. However, in the case of ENMs, the audience expressed some concerns. These concerns were centered around the fact that for the production of common nanomaterial-based products, most production processes are subdivided across different companies and ENM-related information does not propagate through the supply chain. This is primarily as there is no obligation to report as long as the substance does not exceed a threshold that would require the industry to label their use. Dr. Claire Skentelbery (NIA) also added that there is currently a trust deficit due to the lack of communication along the production chain of ENMs and ENM-containing products. Therefore, there is a need for better communication and collaboration between the producers and the regulators. Following the thorough discussion, panelists and participants agreed that the available regulatory frameworks, e.g., REACH, could be evolved for the communication of nanomaterials hazards between different actors. This consequently also means that national regulations have to be harmonized so that producers have concrete guidance for the data generation to achieve regulatory requirements. Furthermore, when suitable communication channels are established, the labeling of ENMs is also an effective means of disseminating the required regulatory information.

The panel reached consensus with the audience that, as expressed by Alain Pardon (Imec), the concern over missing information is warranted. Moreover, if such information for a particular nanoform is present it must be made readily available. That is, it must be incorporated into the safety data sheets, as principal risk communication tools, so that this information becomes immediately available for use in risk assessments. Additionally, labelling of raw materials has to be considered so that different risks become clear for the workers. On the other hand, labelling should not be implemented for all nanomaterial-containing products since it may lead to the public impression that all products bearing nanomaterial labels are hazardous.

## Concluding Remarks

Nanoelectronics was selected as an impactful use case for risk assessment approaches, due to its rapid innovation cycle and culture of high-profile health, safety, risk, and quality management. Moreover, nanoelectronics is one of the key enablers for industrial development, which was also recognized by the European Commission, who designates nanoelectronics among the priority action lines of European industrial policy (24). Therefore, risk management paradigms adopted by the nanoelectronics industry can have widespread economic impacts along its global supply chain. The semiconductor industry is using a growing variety of materials as companies seek to further improve device performance to meet increasing market demand in a constant process of innovation. At present, there are more than 200 chemical compounds, including elements such as silicon, germanium, copper, gold, hafnium, indium, and many others, which are present in most computers and mobile phone chips. In many manufacturing processes, the use of ENMs (15) enables superior yields and performance, a strong competitive driver for their further use.

In conclusion, nanotechnology includes a growing number of industrial applications, with a significant direct economic impact. As such, consumer and environmental exposure to nanomaterials is expected to grow. In view of the economic importance and wide application of nanotechnology, it is very important to foster an open culture of communication of identified hazards and risks to maximize the potential of nanomaterials and remove the overly cautious approach that acts as a bottleneck for the uptake of nanomaterials in products and processes. The workshop identified some key challenges and outlined data gaps which may impede this vision, including the lack of reliable data on nanoform toxicity. Such a gap inadvertently leads to uncertainty in regulations, and it is necessary to identify how much uncertainty is acceptable in view of the direct cost and impact on human health and the environment. Second, risk assessment methods for nanomaterials are far from mature, which makes their application difficult in an industrial setting. Accordingly, innovation guided by the safe-by-design principle must be aided by ongoing and future regulatory research. Finally, it is important to identify appropriate sources of information and communication channels for all actors along the supply chain, including the general public.

The transition from traditional risk assessment toward risk governance which implies moving from hard laws to soft laws is an inevitable trend which is emerging worldwide (25). Some of the issues, underlined in the workshop has been taken up by the newly funded projects, focusing on the risk governance, for example Gov4Nano (www.gov4nano.eu). The caLIBRAte project referenced within the workshop report has now been completed and the Nano Risk Governance Portal is launched and free to access at nanoriskgov-portal.org. The H2020 programme funds three ongoing projects focused on developing the European Risk Governance Council that would provide independent advisory services of importance to industry in general and specifically to the semiconductor industry. The latest development in the field is linking risk governance with designing safe products. Most recently, H2020 funded four consortia to work specifically on nanosafety issues. Similar discussions are ongoing within the USA National Nanotechnology Initiative, where the emphasis is also being shifted toward governance and safety-by-design.

The publication of the REACH Annex amendments in 2019, in which requirements for nanoforms are described, is a major step toward increased regulatory guidance. Nevertheless, the implementation will take many years to fully generate data for increased industrial confidence. Furthermore, risk assessment methods for nanomaterials are far from mature, which makes their application difficult in an industrial setting. Therefore, innovation guided by the safe-by-design principle must be aided by ongoing and future regulatory research. Finally, it is important to identify appropriate sources of information and communication channels for all actors along the supply chain, including the general public. Another welcome development toward providing more information to both consumers and down-stream users has been the publication of the eNanoMapper database through the European Observatory of NanoMaterials (EUON) at euon.echa.europa.eu. In addition, the nanoCommons project, hosted at www.nanocommons.eu, is now active and is providing substantial progress in creating a nanosafety knowledge infrastructure.

## Author Contributions

CS and IL participated in the workshop panels. YC and VC took notes on the discussions. DP and CS co-organized the workshop. PW expanded the notes and took account of the workshop recording. All authors contributed to the manuscript writing.

## Conflict of Interest

The authors declare that the research was conducted in the absence of any commercial or financial relationships that could be construed as a potential conflict of interest.

## Footnotes

^1^https://euon.echa.europa.eu/enanomapper.

## References

[1] EUR-LEX. Regulation (EC) No 1907/2006 of the European Parliament of the Council of 18 December 2006. Concerning the Registration, Evaluation, Authorisation Restriction of Chemicals (REACH), Establishing a European Chemicals Agency, Amending Directive 1999/45/EC Repealing Council Regulation (EEC) No 793/93 Commission Regulation (EC) No 1488/94 as Well as Council Directive 76/769/EEC Commission Directives 91/155/EEC, 93/67/EEC, 93/105/EC 2000/21/EC. (2006). Available online at: httpseur-Lexeuropaeulegal-ContentENTXTuriCELEX3A02006R1907-20140410

[2] Dekkers S Oomen AG Bleeker EAJ Vandebriel RJ Micheletti C Cabellos J. Towards a nanospecific approach for risk assessment. Regul Toxicol Pharmacol. (2016) 80:46–59. 10.1016/j.yrtph.2016.05.037 27255696

[3] Jiménez AS Varet J Poland C Fern GJ Hankin SM Tongeren. A comparison of control banding tools for nanomaterials. J Occup Environ Hyg. (2016) 13:936–49. 10.1080/15459624.2016.1200191 27314531

[4] Isigonis P Hristozov D Benighaus C Giubilato E Grieger K Pizzol L. Risk governance of nanomaterials: review of criteria and tools for risk communication, evaluation, and mitigation. Nanomaterials. (2019) 9:696. 10.3390/nano9050696 31060250 PMC6566360

[5] Watjanatepin P Prodano D Tools for assessment of occupational health risks of some engineered nanoparticles and carbon materials used in semiconductor applications. In: Occupational Health and Safety - A Multi-Regional Perspective. London (2018). 10.5772/intechopen.76567

[6] Trump BD Hristozov D Malloy T Linko I Risk associated with engineered nanomaterials: different tools for different ways to govern. Nano Today. (2018) 21:9–13. 10.1016/j.nantod.2018.03.002

[7] Krug H Bohmer N Kühnel D Marquardt C Nau K Steinbach. The DaNa2.0 knowledge base nanomaterials—an important measure accompanying nanomaterials development. Nanomaterials. (2018) 8:204. 10.3390/nano8040204 29596351 PMC5923534

[8] NIOSH Continuing to Protect the Nanotechnology Workforce: NIOSH Nanotechnology Research Plan for 2018−2025 US Department of Health and Human Services, Public Health Service, Centers for Disease Control and Prevention, National Institute for Occupational Safety and Health, Cincinnati, OH (2019).

[9] Krug HF. Nanosafety research-are we on the right track? Angew Chem Int Ed. (2014) 53:12304–19. 10.1002/anie.201403367 25302857

[10] Hristozov DR Gottardo S Critto A Marcomini A. Risk assessment of engineered nanomaterials: a review of available data and approaches from a regulatory perspective. Nanotoxicology. (2012) 6:880–98. 10.3109/17435390.2011.626534 22229953

[11] Linkov I Anklam E Collier ZA DiMase D Renn O Risk-based standards: integrating top–down and bottom–up approaches. Environ Syst Decis. (2014) 34:134–7. 10.1007/s10669-014-9488-3

[12] ISO Nanotechnologies - Occupational Risk Management Applied to Engineered Nanomaterials. Part 2: Use of the Control Banding Approach. ISO/TS12901-2. ISO (2014).

[13] Liguori B Hansen SF Baun A Jensen KA Control banding tools for occupational exposure assessment of nanomaterials — ready for use in a regulatory context? NanoImpact. (2016) 2:1–17. 10.1016/j.impact.2016.04.002

[14] Rycroft T Larkin S Ganin A Thomas T Matheson J Van Grack T A framework and pilot tool for the risk-based prioritization and grouping of nano-enabled consumer products. Environ Sci Nano. (2019) 6:356–65. 10.1039/C8EN00848E

[15] Belde P Durand C Jank M Maillet J-C Moclair F A List of Nanomaterials Currently Used in the Semiconductor Sector to be Considered Within Nanostreem. Geneva (2016). 10.5281/zenodo.804437

[16] Belde P Maillet J-C Moclair F Morelli A Prodanov A List of Associated Tasks, Activities and Operations Where Exposure Might Occur. Geneva (2016). 10.5281/zenodo.804442

[17] Prodanov D Belde P Geerts L L'Allain C Feber ML Moclair F Three-tiered risk assessment for engineered nanomaterials. A use case for the semiconductor industry. J Phys Conf Ser. (2019) 1323:012010 10.1088/1742-6596/1323/1/012010

[18] Fadeel B Farcal L Hardy B Vázquez-Campos S Hristozov D Marcomini A. Advanced tools for the safety assessment of nanomaterials. Nat Nanotechnol. (2018) 13:537–43. 10.1038/s41565-018-0185-0 29980781

[19] ISO Nanotechnologies - Health and Safety Practices in Occupational Settings Relevant to Nanotechnologies. Geneva: ISO (2008).

[20] Le Feber M Prodanov D Zimmermann E Identification of the Most Appropriate Risk Assessment Methodologies for Use in the Semiconductor Industry. Geneva (2017). 10.5281/zenodo.1188143

[21] Schinkel J Warren N Fransman W van Tongeren M McDonnell P Voogd E. Advanced REACH Tool (ART): calibration of the mechanistic model. J Environ Monit. (2011) 13:1374–82. 10.1039/c1em00007a 21403945

[22] Boccuni F Ferrante R Tombolini F Pingue P Porcari A Iavicoli S Workers' exposure to nano-objects in R&D laboratories: an integrated risk management and communication approach. Saf. Sci. (2020) 129:104793 10.1016/j.ssci.2020.104793

[23] Linkov I Steevens J Adlakha-Hutcheon G Bennett E Chappell M Colvin V. Emerging methods tools for environmental risk assessment, decision-making, policy for nanomaterials: summary of NATO advanced research workshop. J Nanoparticle Res. (2009). 11:513–27. 10.1007/s11051-008-9514-9 19655050 PMC2720173

[24] EC Communication from the Commission to the European Parliament, the Council, the European Economic and Social Committee and the Committee of the Regions - “Preparing for Our Future: Developing a Common Strategy for Key Enabling Technologies in the EU” SEC 20091257. Brussels (2009).

[25] Linkov I Trump BD Anklam E Berube D Boisseasu P Cummings C Comparative, collaborative, and integrative risk governance for emerging technologies. Environ Syst Decis. (2018) 38:170–6. 10.1007/s10669-018-9686-5 PMC10569133 37829286

