# Supplementary material for: Workshop Report: Governance of Emerging Nanotechnology Risks in the Semiconductor Industry
Source: Front Public Health. 2020 Jul 7;8:275. doi: 10.3389/fpubh.2020.00275 (PMC7358517; doi:10.3389/fpubh.2020.00275)
Supplement: Supplementary file 1 [file Data_Sheet_1.docx]

# Appendix I

#### A detailed list of identified challenges

- The exponential growth of amount ENM in the market all over the world.
- Safety knowledge gap related to exposure still present.
- Lack of resources makes the regulatory approaches challenging.
- Reliable prediction of hazards and risks from physicochemical data.
- Lack of material and toxicity data.
- Many databases are not available for public use.
- The conventional risk assessment methodology is not adequate for newly developed materials in the market.
- Risk analysis is still technically and methodologically limited.
- Risk acceptance is strongly dependent on the understanding of the risk.
- Exposure scenarios need to be developed specifically case by case (for each production type)
- Which ENMs should be considered when labelling consumer products?

#### A detailed list of identified gaps

- Accessible databases and libraries incomplete. Difficult access to data for the manufacturers
- Exposure data and models from stakeholders incomplete
- Consensus on the hazard metric
- Exposure scenarios incomplete
- ENMs grouping unavailable. Grouping nanomaterials is an important means that may enable faster ENM risk assessment
- Risk management during the process of innovation of new products and new technologies.
- Calibration of exposure tools for ENMs.
- Professional nano-safety training unavailable
